# Kefir peptides enhance neuron viability in a in vitro model of β‐amyloid aggregation

**DOI:** 10.1002/alz70859_103660

**Published:** 2025-12-26

**Authors:** Fernanda Araújo do Prado mascarenhas, Ana Carolina Costa Santos, Tamiris Sabrina Rodrigues, Serena Mares Malta, Lucas Matos Martins Bernardes, Matheus Henrique Silva, Renata Graciele Zanon, Carlos Ueira‐Vieira, Ana Paula Mendes‐Silva

**Affiliations:** ^1^ AV. Amazonas, 1777, bloco2A sala10, Uberlândia, Minas Gerais Brazil; ^2^ Universidade Federal de Uberlândia, Uberlândia, Minas Gerais Brazil; ^3^ University of Saskatchewan, Saskatoon, SK Canada

## Abstract

**Background:**

Alzheimer's disease (AD) is a progressive neurodegenerative disorder and the most common cause of dementia in older adults. The amyloidogenic hypothesis, the leading theory explaining this condition, describes the extracellular buildup of amyloid beta (Aβ) peptides. Kefir, a fermented dairy product, has gained prominence in the literature due to its health benefits, including modulating the intestinal microbiota. This modulation is particularly relevant as the gut‐brain axis plays a critical role in influencing neuronal survival and inflammatory processes. In this study, we evaluate the effect of four kefir peptides with high homology to the human Aβ 1‐42 peptide domain on neuron viability using an in vitro model of Aβ aggregation.

**Methods:**

Four kefir peptides (PW, M11, M20, and M25) were selected from our previous in silico findings and synthesized. We used the human neuronal cell line, SH‐SY5Y, to standardize two experimental approaches to evaluate the effect of kefir peptides on cell viability before and after senile plaque formation. Amyloid beta peptide [886 μM] was diluted to 1 μM and followed by two models, one of incubation together with kefir peptides and then added to the cells, and another of incubation and addition to the cells, promoting senile plaque formation, to later be treated with kefir peptides. After the treatment period, cell viability was evaluated by the AlamarBlue assay (Invitrogen®) and oxidative stress by DCFH‐DA staining (Cayman Chemical®). Statistical analysis by Student's t‐test (GraphPad Prism v9.0) (*p* ≤ 0.05).

**Results:**

Peptides M11, M20 and M25 significantly increased neuronal viability when administered simultaneously with amyloid beta before senile plaque formation, while PW did not show a significant effect. Regarding viability after senile plaque formation, all peptides studied showed an increase when compared to the control groups. Both peptides also showed potential to attenuate intracellular oxidative stress when administered before or after senile plaque formation.

**Conclusions:**

In general, kefir peptides increased neuronal viability and attenuated intracellular oxidative stress before and after senile plaque formation. These findings suggest that kefir peptides may offer neuroprotective benefits in the context of Alzheimer's disease.

